# Natural Killer Cells in Asthma

**DOI:** 10.3389/fimmu.2013.00159

**Published:** 2013-06-21

**Authors:** Khalil Karimi, Paul Forsythe

**Affiliations:** ^1^Institut für Experimentelle Immunologie und Hepatologie, Universitätsklinikum Hamburg-Eppendorf, Hamburg, Germany; ^2^Department of Medicine, Brain-Body Institute, McMaster University, Hamilton, ON, Canada; ^3^Department of Medicine, Firestone Institute for Respiratory Health, McMaster University, Hamilton, ON, Canada

**Keywords:** natural killer cells, asthma, airway inflammation, asthma exacerbation, therapeutic potential of NK cells

## Abstract

The worldwide prevalence, morbidity, and mortality of asthma have dramatically increased over the last few decades and there is a clear need to identify new effective therapeutic and prophylactic strategies. Despite high numbers of NK cells in the lung and their ability to generate a variety of immunomodulatory mediators, the potential of NK cells as therapeutic targets in allergic airway disease has been largely overlooked. The fact that IgE, acting through FcγRIII, can activate NK cells resulting in cytokine/chemokine production implies that NK cells may contribute to IgE-mediated allergic responses. Indeed, current evidence suggests that NK cells can promote allergic airway responses during sensitization and ongoing inflammation. In animal models, increased NK cells are observed in the lung following antigen challenge and depletion of the cells before immunization inhibits allergic airway inflammation. Moreover, in asthmatics, NK cell phenotype is altered and may contribute to the promotion of a pro-inflammatory Th2-type environment. Conversely, driving NK cells toward an IFN-γ-secreting phenotype can reduce features of the allergic airway response in animal models. However, we have limited knowledge of the signals that drive the development of distinct subsets and functional phenotypes of NK cells in the lung and thus the role and therapeutic potential of NK cells in the allergic airway remains unclear. Here we review the potentially diverse role of NK cells in allergic airway disease, identify gaps in current knowledge, and discuss the potential of modulating NK cell function as a treatment strategy in asthma.

## Asthma

Asthma is a chronic airway disease, whose symptoms include wheezing, chest tightness, breathlessness, and coughing with variable and often reversible airway obstruction together with airway hyper-responsiveness (Holgate, 2011b).

Asthma generally develops in childhood and is associated with sensitization of the airways to aeroallergens such as house dust mites, cockroaches, animal dander, fungi, and pollens (Holgate, 2011a). In most cases, this occurs through the selective expansion of T lymphocytes that secrete an array of largely Th2-type cytokines. These cytokines drive the characteristic events of airway inflammation central to disease pathophysiology in allergic asthma. Such events include: promotion of Th2 cell survival, B cell isotype switching to IgE synthesis, mast-cell differentiation and maturation, basophil recruitment, and eosinophil maturation and survival (Corrigan et al., 1988; Humbles et al., 2004; Kay, 2005, 2006; Holgate, 2011b). Variable airflow obstruction and associated airway hyper-responsiveness are driven both by the release of potent inflammatory mediators and through remodeling of the airway wall. As the severity of disease increases, the airway becomes more susceptible to environmental insults such as biologically active allergens, viruses, and air pollutants. Damage caused by these insults is further enhanced by an altered repair response leading to mucus cell metaplasia, smooth muscle proliferation, increased innervation, and fibrosis (Tang et al., 2006; Folli et al., 2008; Bara et al., 2010).

While adaptive immune responses have been well studied in regard to asthma, in recent years, there has been increased awareness and interest in the role of innate immune responses in regulating susceptibility to, and severity of, asthma. In particular, the role of innate responses to respiratory viral infections in driving allergic asthma and the potential for certain bacterial signals to protect against the disease have come under intense investigation (reviewed in von Mutius and Vercelli, 2010; Holgate, 2012; Holtzman, 2012).

## Natural Killer Cells and Lung Inflammation

NK cells are the key to the natural defenses of the body (Cooper et al., 2009) and have been detected in virtually all species from invertebrates to mammals. As components of the innate immune response, NK cells do not require previous sensitization to instigate their action (Kiessling et al., 1975; Forsythe et al., 2007). NK cells mediate cellular toxicity (Leavy, 2012) and release chemokines and cytokines involved in combating tumors (Karimi et al., 2008), viral infections (Berahovich et al., 2006), parasites (Evans and Jaso-Friedmann, 1993), and bacteria (Ashkar et al., 2009). Uterine NK cells also release mediators, such as vascular endothelial growth factor (VEGF) and placental growth factor (PGF), which play a major role in the vascularization of implanting embryos during pregnancy (Ashkar et al., 2000; Kalkunte et al., 2008).

The function of NK cells is regulated by a large repertoire of inhibitory and activating receptors (Raulet et al., 2001; Lanier, 2008). One particular activating receptor, NKp46, found on a rare T-cell population (Narni-Mancinelli et al., 2011) and a subset of gut innate lymphoid cells (ILCs) (Tomasello et al., 2012), is considered a unifying surface marker of NK cells across all species (Walzer et al., 2007). However, in humans, NK cells have traditionally been defined as cells that lack the T-cell receptor CD3 but express CD56 with or without CD16 (Milush et al., 2009). In mice, NK cells are defined as CD3^−^ NK1.1^+^ or DX5^+^ cells (Wingett and Nielson, 2003).

NK cell-activating receptors include the natural cytotoxicity receptors (NCRs), such as NKp46 and NKp44, the Fc receptor CD16, and NKG2D. The ligands for NK cell-activating receptors include both host and pathogen glycoproteins. Thus, NKG2D recognizes the stress-induced ligand MHC class I polypeptide-related sequence A (MICA), while NKp46 and NKp44 can directly bind influenza hemagglutinin, a response that is key to the role of NK cells in protecting against infection by the virus (Arnon et al., 2001; Mandelboim et al., 2001).

It is also important that NK cells remain tolerant of healthy tissue, and as such they express receptors that can prevent cell activation. Inhibitory receptors, such as killer immunoglobulin-like receptors (KIRs) and the NKG2A:CD94 dimer, generally recognize classical and non-classical class I major histocompatibility complex (MHC) molecules (Colonna et al., 1993; Valiante et al., 1997; Boyington et al., 2000; Culley, 2009).

NK cells develop in the bone marrow and migrate to the blood as circulating cells, or become resident cells in tissues such as the liver, spleen, lymph nodes, uterus, and lungs. NK cell recruitment to tissues is under the control of the chemokine receptors CCR2, CCR5, CX3CR1, and CXCR3 (Di Santo, 2006) and migration can occur in response to infectious (Salazar-Mather et al., 1998; Zeng et al., 2003) or allergic inflammation (von Bubnoff et al., 2010). The relatively high frequency as well as total number of NK cells in the lung compared to other organs (Gregoire et al., 2007; Culley, 2009) supports a prominent role for these cells in the immunology of the airways.

Innate immune cells in the lung have the job of defending the organ’s vital function against the constant barrage of exposures to environmental antigens, commensal organisms, and potential pathogens. The role of NK cells in protecting against respiratory infection by fungi, bacteria, and viruses [including influenza and respiratory syncytial virus (RSV)] has been well described in mouse models (reviewed in Culley, 2009).

NK cells control viral infections by multiple mechanisms including up-regulating the expression of NKG2D (Lodoen et al., 2003), IFN signaling (Trinchieri et al., 1978), and cytokine and chemokine secretion (Vujanovic et al., 2010), which bring about cytolysis of the virally infected cells (Malhotra and Shanker, 2011). Following influenza infection, NK cells are increased in the lungs (Gazit et al., 2006; Nakamura et al., 2010), activate CD8+ T-cell effector functions (Qian et al., 1998), and modulate pulmonary inflammation (Abdul-Careem et al., 2012). NK cells are responsible for lung injury during respiratory virus infection either alone (Yasui et al., 2004; Li et al., 2012) or in combination with IL-18 (Harker et al., 2010).

**Figure 1:**
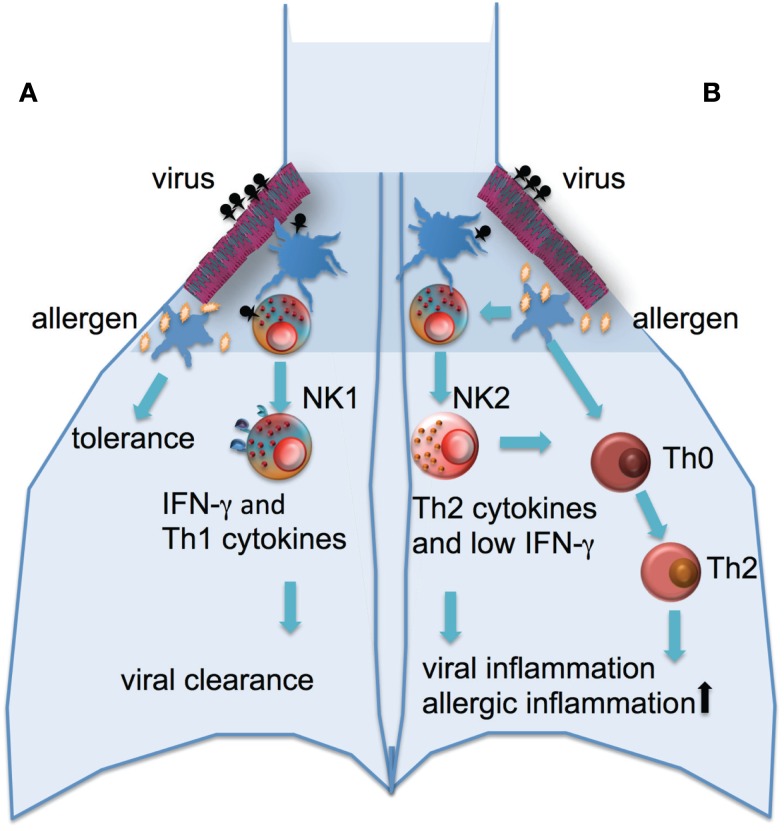
**Potential role of NK cells in the development of allergic airway disease**. **(A)** A robust NK cell response to respiratory viral infection with high levels of IFNγ production and cytolytic activity this leads to viral clearance and Th1 environment in the lung and normal tolerance to environmental antigens. **(B)** An impaired response to viral infection with low IFNγ and cytolytic activity and higher levels of Th2-type cytokines released from NK cells leads to increased inflammation and viral shedding, the recruitment of immature DCs, allergic sensitization with subsequent inflammation.

The contribution of NK cells to the defense against viral infection may also afford the cells a pivotal role in regulating the pathogenesis of asthma. Infection with rhinoviruses is not only the major cause of asthma exacerbations, there is also extensive evidence of a relationship between the development of asthma and severe lower respiratory infections early in life (Jackson et al., 2008, 2012). In those genetically at risk of asthma, rhinovirus-induced wheezing in the first 3 years of life is the greatest risk factor for developing asthma at 6 years of age (Jackson et al., 2008). Whether there is a causal relationship between viruses and asthma development or whether infection and asthma have shared risk factors is not entirely clear, but it has been suggested that the release of inflammatory products and enhanced viral shedding provide a strong stimulus for recruitment of immature DCs and their priming for allergen sensitization (Holgate et al., 2009; Kumar and Grayson, 2009). It has been demonstrated that absence of NK cells during primary RSV infection of mice resulted in the suppression of IFNγ production, as well as the development of an RSV specific Th2 response leading to an allergic airway response to bystander antigens (Kaiko et al., 2010). This development of an allergic airway response appeared to be due to the loss of IFNγ production in the absence of NK cells and subsequent increased production of IL-25 by airway epithelial cells (Kaiko et al., 2010). Thus, NK cell function may be a major determinant of the development of viral associated asthma (Figure 1).

Mouse models indicate NK cells may also have an important role in the allergic airway response beyond a relationship with viral infection. NK cells have been demonstrated to contribute to the initiation and development of T-cell mediated allergic airway inflammation (Korsgren et al., 1999; Wingett and Nielson, 2003; Ple et al., 2010). NK cell recruitment and accumulation at the site of immunization and generation of a panel of cytokines which are involved in the pathogenesis of allergic inflammation suggest that these cells play a critical role at several steps in the development of the acquired immune response to allergens. Additionally, NK cells have been demonstrated to produce IFN-γ, TNF-α, GM-CSF, and MIP-1α upon IgE stimulation and exhibit cytotoxicity against IgE-coated target cells in an FcγRIII-dependent manner (Arase et al., 2003). The fact that NK cells can be activated with IgE through FcγRIII resulting in cytokine/chemokine production implies that NK cells may contribute to IgE-mediated allergic responses.

Early clinical observations indicated that NK activity was enhanced in peripheral blood from asthmatic subjects (Timonen and Stenius-Aarniala, 1985; Jira et al., 1988) but decreased immediately following antigen challenge (Jira et al., 1988). It has been suggested that this decrease in NK activity following antigen challenge reflects migration of NK cells from the circulation to lymphoid compartments and the lungs (Jira et al., 1988; Culley, 2009). However, it cannot be excluded that agents in the circulation of challenged allergic individuals may directly inhibit NK activity (Jira et al., 1988). A more recent study (Lin et al., 2003) demonstrated that NK cell frequency was increased in peripheral blood from asthmatic children during acute exacerbation when compared to stable asthmatics. However, during an exacerbation, peripheral blood NK cell expression of adhesion molecules, ICAM-1 and L-selectin, were significantly decreased. Again, it was suggested that during an exacerbation of asthma, NK cells expressing ICAM-1 and L-selectin may selectively migrate into inflamed lung tissues or, alternatively, subsets of NK cells that do not express ICAM-1/L-selectin are expanded. Clearly, further clinical studies are required to characterize the phenotype and function of NK cells in allergic individuals and directly address the fate of the cells following antigen challenge of the airway.

With regard to animal models, Ple et al. (2010) demonstrated that sensitization and airway challenge with OVA increased the number of immature CD27^*low*^CD11b^*low*^ and CD27^*high*^CD11b^*low*^ NK cells in the draining lymph nodes of the mouse lung. The NK cells in the lung lymph nodes expressed higher levels of CD86, suggesting that cross-talk between these cells and CD4+ T-cells may be important, potentially increasing Th2 cytokine production. Depletion of NK cells following OVA challenge lead to a dramatic decrease in eosinophil influx to the airway in response to antigen challenge (Ple et al., 2010). In contrast, a previous study demonstrated that eosinophilic airway inflammation was reduced when NK cells were depleted prior to sensitization, while depletion following sensitization, just prior to antigen challenge, had no significant effect (Korsgren et al., 1999). Similarly, depletion of NK cells prior to sensitization lead to a decrease in serum IgE while depletion following sensitization did not alter IgE levels (Korsgren et al., 1999). The reasons for the differences between studies in relation to the effect of depleting NK cells following the sensitization phase are unclear. However, it is suggested that NK cells may influence multiple pathways during the progression of asthma. NK cells may be involved in the development of a Th2 response but do not appear to influence this response once it is established and may instead be involved in recruiting eosinophils through the release of IL-5. An effect that has been described following IgE-mediated activation of NK cells (Arase et al., 2003).

While there is good evidence for a pro-inflammatory and asthma-promoting role of NK cells, there are also indications that these cells are involved in the resolution of acute allergic airway inflammation. Haworth et al. (2011) demonstrated that during clearance of eosinophils and T-cells from the airway following an inflammatory response there are increased numbers of activated NK cells. Depletion of NK cells delayed the resolution of both eosinophils and CD4+ T-cells from the lungs. Furthermore, neutralizing CXCR3 on NK cells impeded the NK cells’ ability to reach lung lymph nodes and also delayed resolution (Haworth et al., 2011) of inflammation.

With existing evidence suggesting that NK cells are involved in both the promotion and inhibition of allergic lung inflammation, it will be important to determine the mechanisms underlying these contrasting roles and to determine how the shift in cell function, from promoting to resolving inflammation, occurs.

## NK Subtypes and Asthma

In addition to the rapid production of IFN-γ, NK cells have the potential to generate a range of other cytokines, including IL-5, IL-8, IL-10, IL-22, IFN-γ, TNF, GM-CSF, MCP-1, MIP-1α, and RANTES (Michel et al., 2010). Indeed, based on the profile of cytokine production, NK cells can be divided into different functional subsets, analogous to T-cell subsets (Th1/Th17, Th2, and T regulatory cells). Thus, NK1/NK17 cells produce IFN-γ and IL-17 (Pandya et al., 2011), whereas NK2 cells produce IL-4, IL-5, and IL-13 (Loza and Perussia, 2001; Katsumoto et al., 2004), and NKreg secrete TGFβ and IL-10 (Lang et al., 2012). It has been observed that NK cells grown in IL-12 produce IL-10 and IFN-γ and express high levels of Fas, whereas NK cells grown in IL-4 are IL-5-/IL-13-producing cells, which express low levels of Fas (Peritt et al., 1998). Additionally, phenotypic and functional characterization of the NK1 and NK2 cell subsets revealed that human NK2 cells are CD56^*low*^CCR7^−^, whereas human NK1 cells are CD56^*hi*^CCR7^+^. The addition of Cho-kyung-jong-ok-tang (CKJOT), a traditional Korean herbal formula, to NK cell differentiation cultures suppressed the production of IFN-γ and down-regulated T-bet transcription in NK1 subset, while enhancing the release of IL-5 and up-regulating Th2 linked transcription factors STAT6 and GATA3 in NK2 subset (Lee et al., 2011). Although most evidence suggests that NK2 cells arise from NK1 cells, the possibility of both cells originating from a common precursor (an immature NK cell) has been suggested (Herberman et al., 1975).

*In vivo*, microenvironmental factors can condition the generation and function of distinct NK cell subsets (Di Santo, 2008). Wei et al. (2005) identified that IL-4^+^ NK cell frequency was increased and IFN-γ^+^ NK cell frequency was decreased in patients with asthma compared to healthy individuals. Furthermore, successful treatment of the asthmatics leading to reduced symptoms was associated with a reversal of the shift toward IL-4 expressing NK cells. Such observations lead the authors to suggest that an NK2 cell subset is involved in the pathogenesis of asthma. Similarly, Aktas et al. (2005) demonstrated that NK cells from atopic patients spontaneously released higher amounts of IL-4, IL-5, IL-13, and IFN-γ compared to healthy donors. Furthermore, stimulation of NK cells from allergic individuals, but not control subjects, lead to a significant increase in IL-5- and IL-13-producing subsets, again suggesting that NK2 cells are involved in the unbalanced cytokine profile of allergic inflammation. More recently, it was observed that the CD56^*high*^ NK subset is decreased in asthmatic patients (Scordamaglia et al., 2008). Furthermore, asthmatics exhibited reduced NK cell-mediated IFN-γ production in response to DC together with decreased capability of promoting DC maturation and/or killing immature DC (Scordamaglia et al., 2008). These findings suggest that, in addition to enhanced NK2 cell frequency, allergic asthma is associated with a decreased capability of NK cells to promote and sustain Th1 responses. The association of NK2 bias with asthma exacerbation might indicate that immunologic interventions preventing such an NK bias might benefit patients with asthma (Ozdemir, 2005). Additionally, given the ability of NK cells to influence adaptive immune responses, the differential development of distinct NK phenotypes in asthma may be an important influence in disease pathogenesis.

## NK Cell Interactions with Other Immune Cells

The regulation of NK phenotype in the normal and asthmatic lungs will depend on the microenvironment and interactions with other cell types. Indeed, survival of NK cells within the airway mucosa may depend on lung epithelial cell-derived IL-15 (Ge et al., 2004). Dendritic cells and macrophages (Laskin et al., 2001) are among lung resident cells that form synapses (Nedvetzki et al., 2007; Wehner et al., 2011) with NK cells. Such interactions lead to increased generation of NK-derived cytokines and effector molecules that are involved in pulmonary immunity and have the potential to influence allergic disease severity. Resting NK cells respond rapidly to cytokine signals that are delivered by myeloid cells (Chenoweth et al., 2012). DC-derived type I interferon (Strowig et al., 2008), IL-12 (Watford et al., 2003), and IL-15 (Lucas et al., 2007) can activate NK cells, leading to secretion of granular contents and IFN-γ (Gill et al., 2009). DC can attract NK cells via CCR5 (Van Elssen et al., 2010) or CXCR3 (Persson and Chambers, 2010) and prime the cells to exhibit higher cytotoxicity and IFNγ production in response to inflammatory or infectious stimuli (Walzer and Vivier, 2011). IFNγ production from NK cells can, in turn, polarize CD4^+^ T-cells toward a Th1 response (Morandi et al., 2006). Conversely, NK cells can attract immature DCs to lymph nodes, likely through the production of chemokines such as CXCL1, CCL3, CCL4, and CCL5 (Eberlein et al., 2010; Walzer and Vivier, 2011). NK cells activated with IL-12 can kill immature DCs while NK cells activated with IL-4 do not perform this function (Voehringer et al., 2002). In addition to interactions with antigen presenting cells, activated T-cells induce secretion of IFN-γ from human NK cells, an effect mediated by the action of IL-2 on high-affinity receptors constitutively expressed on the CD56^*high*^ NK subset (Fehniger et al., 2003). Conversely, NK cell activity can be suppressed by TGF-β derived from CD4^+^CD25^+^ regulatory T-cells (Barao et al., 2006). Mast cells can also modulate NK cell activity. Lipopolysaccharide activated mast cells can significantly enhance IFN-γ production through a cell contact dependent mechanism that depends, in part, on OX40 ligand expressed by the mast cell (Vosskuhl et al., 2010).

It appears that the cytokine profile and cellular makeup of the microenvironment leads to the development of specific NK subtypes. Similarly, particular combinations of chemokines attract selected NK subtypes to sites of inflammation. As cellular cytokine and chemokine profiles do change over the course of an inflammatory response, this may lead to the sequential transformation of NK cells from a pro-inflammatory to a pro-resolution subtype (Walzer and Vivier, 2011). In the case of asthma, defects or disruption of this process could lead to prolonged and more severe inflammation leading to airway damage.

## The Therapeutic Potential of NK Cells in Asthma

Evidence indicates that NK cells have a disease-promoting or disease-controlling role in allergic asthma via their contribution to allergen-specific immune suppression, allergen-specific Th1 cell generation, and IgE production (Erten et al., 2008). Thus, ongoing studies should focus on NK cell subsets and their interactions with other immune cells during sensitization and the inflammatory response to antigen challenge in lung mucosa. Such studies will provide powerful insights into the therapeutic potential of NK cells.

In the past decade, the adoptive transfer of *in vitro*-cultured and -activated human NK cells has been applied toward the treatment of certain cancers (Gill et al., 2009). Although such adoptive transfers are generally thought to be transient due to a relatively short lifespan of NK cells, Sun et al. (2009) demonstrated that after mouse *Cytomegalovirus* infection, long-lived NK cells are generated and exist for months in lymphoid and non-lymphoid tissue sites. These long-lasting NK cells can mount protective secondary responses when the virus is reencountered (Sun et al., 2009). More recently, the same researchers demonstrated that adoptive transfer of mature NK cells into lymphopenic mice resulted in the generation of a long-lived cell population (Sun et al., 2011). Such homeostasis-driven NK cells maintained activity in the recipient for longer than 6 months. These findings have led to suggestions that methods may be developed, which allow for the adoptive transfer of long-lasting and/or self-renewing NK cells that persist for months to years and may provide potent therapeutic strategies for cancers or viral infection (Sun and Lanier, 2011; Sun et al., 2011). However, the potential detrimental effects of having a persistent population of activated NK cells, for example, the risk of graft versus host disease, remain to be explored.

It may be possible that a similar adoptive transfer approach could be extended toward asthma therapy. *In vitro* expansion and adoptive transfer of a desired subset of NK cells, or endogenous expansion of NK cells *in vivo* with the use of appropriate cytokines, could represent therapeutic approaches to resolve allergic inflammation. Options could include inducing NKreg mediated suppression or enhancing NK1 cells to balance the skew toward type 2 immune responses in allergic asthma. However, the ability to maintain the transferred cells phenotype in the face of the cytokine milieu and cellular microenvironment of the recipient is uncertain.

Another, perhaps more readily achievable, approach to modulating NK phenotype and function could be to take advantage of the cell response to commensal microbes. In mouse studies, administration of certain lactic acid bacteria (LAB) can protect against respiratory pathogens (Hori et al., 2002; Yasui et al., 2004; Harata et al., 2010; Izumo et al., 2010; Kawashima et al., 2011; Kawase et al., 2012). These protective effects do not require direct exposure of the organism to the airway mucosa. LAB can attenuate airway infection through an interaction with the gut associated lymphoid tissue, such as Peyer’s patches, leading to indirect enhancement of respiratory immunity. In a number of studies the protective effects of both intranasal and oral bacteria have been associated with up-regulation of NK cell activity in the airway mucosa (Yasui et al., 2004; Harata et al., 2010; Izumo et al., 2010; Kawase et al., 2012). A recent study demonstrated that oral administration of *Lactobacillus plantarum* significantly reduced lung viral titer in a mouse model of influenza infection. The reduced viral titer was associated with increases in pulmonary IFN-α, IL-12, and IFN-γ production and a marked up-regulation of splenic NK activity (Takeda et al., 2011). The increases in pulmonary IFN-γ and IL-12 production also correlated with augmentation of IFN-γ and IL-12 receptor mRNA expression in Peyer’s patches, suggesting that the bacteria can elicit immunomodulatory effects in the lung by stimulating intestinal immunity. Koizumi et al. (2008) demonstrated that feeding mice with *L. pentosus* significantly enhanced NK activity of spleen cells and induced NK1.1-positive NK and NK T-cells to produce IFN-γ. The increase in IFN-γ production did not occur through direct action of *L. pentosus* on NK cells but was dependent on IL-12 produced by CD11c^+^ DCs following a toll-like receptor (TLR) 2- and/or TLR4-dependent interaction between the DC and the bacteria. Strains of LAB differ greatly in their ability to induce high levels of IL-12 in human DCs and consequently DC-dependent IFN-γ production by NK cells (Koizumi et al., 2008). LAB treatment has also been demonstrated to attenuate allergic responses, repressing IgE production and reducing antigen-induced inflammation (Shida et al., 2002; Fujiwara et al., 2004; Forsythe et al., 2007). There is good evidence that the anti-allergy effects of certain LAB strains is associated with the ability of the bacteria to skew the allergic Th2 response toward a Th1 response via the induction of IL-12 in antigen presenting cells (Shida et al., 1998, 2002; Fujiwara et al., 2004; Pohjavuori et al., 2004; Ongol et al., 2008; Izumo et al., 2010; Kawashima et al., 2011). Given the role of NK cells in establishing a Th1 response, it is tempting to suggest that NK cell-derived IFN-γ released following interaction with DC contributes to the Th1 polarizing effects of certain LAB. Thus, investigation of how specific bacterial signals differentially regulate the development of NK subtypes may aid in the development of microbial-based therapeutic strategies for lung infections and inflammatory diseases, including asthma.

## Conclusion

A better understanding of the mechanisms of action of NK cells in allergic asthma is needed. NK cells appear to contribute to the sensitization phase of an allergen-specific adaptive immune response, to the balance between a Th1 and Th2-type response and also to the resolution of ongoing inflammation. The influence of NK cells on these processes is dependent on the activation state and subtype of the cells that, in turn, is strongly dependent on elements on the lung microenvironment, including commensal organisms, pathogens, and the inflammatory state. Increased evidence of cross-talk between NK cells and other immune cells has raised the possibilities of exploiting this interplay to regulate activation and inhibition of NK cells for immunotherapeutic purposes. Thus, through secretion of cytokines such as IFN-γ or IL-5, NK cells could influence antigen presenting cells, DC maturation, and the eosinophil influx that is associated with allergic airway disease. Cytolytic mechanisms may also contribute to the influence of NK cells in allergic airway disease, with lysis of DCs or macrophages modulating antigen presentation and, in turn, influencing the T-cell response. Questions that remain include: how do various subtypes of NK cells and specific NK-derived cytokines/chemokines contribute to the promotion/cessation of allergic sensitization? What governs the polarization of NK subtypes during the progression of inflammation? and to what degree does disruption of this process contribute to asthma pathogenesis?

With increased knowledge, it may be possible to direct the recruitment and development of specific subtypes of NK cells that will provide protective and/or therapeutic strategies in allergic airway disease.

## Conflict of Interest Statement

The authors declare that the research was conducted in the absence of any commercial or financial relationships that could be construed as a potential conflict of interest.
